# Risk-Sensitivity in Bayesian Sensorimotor Integration

**DOI:** 10.1371/journal.pcbi.1002698

**Published:** 2012-09-27

**Authors:** Jordi Grau-Moya, Pedro A. Ortega, Daniel A. Braun

**Affiliations:** 1 Max Planck Institute for Biological Cybernetics, Tübingen, Germany; 2 Max Planck Institute for Intelligent Systems, Tübingen, Germany; Johns Hopkins University, United States of America

## Abstract

Information processing in the nervous system during sensorimotor tasks with inherent uncertainty has been shown to be consistent with Bayesian integration. Bayes optimal decision-makers are, however, risk-neutral in the sense that they weigh all possibilities based on prior expectation and sensory evidence when they choose the action with highest expected value. In contrast, risk-sensitive decision-makers are sensitive to model uncertainty and bias their decision-making processes when they do inference over unobserved variables. In particular, they allow deviations from their probabilistic model in cases where this model makes imprecise predictions. Here we test for risk-sensitivity in a sensorimotor integration task where subjects exhibit Bayesian information integration when they infer the position of a target from noisy sensory feedback. When introducing a cost associated with subjects' response, we found that subjects exhibited a characteristic bias towards low cost responses when their uncertainty was high. This result is in accordance with risk-sensitive decision-making processes that allow for deviations from Bayes optimal decision-making in the face of uncertainty. Our results suggest that both Bayesian integration and risk-sensitivity are important factors to understand sensorimotor integration in a quantitative fashion.

## Author Summary

Statistically optimal decision-makers use probabilistic predictive models of their environment to achieve their goals. However, in real life such probabilistic models can be wrong or only approximately true, in which case basing decisions exclusively on the statistics of such models can constitute a problematic decision criterion. In contrast, risk-sensitive decision-makers can take model uncertainty into account. They allow deviations from their probabilistic model depending on the quality of the predictions of the model. In particular, they trust their model less if it makes imprecise predictions and bias their decisions towards worst-case or best-case outcomes. Here we designed a sensorimotor task where subjects exhibit Bayesian information integration when they infer the hidden location of a target and they had to decide to make a more or less costly movement. We found that subjects exhibited a bias with respect to the statistically optimal movement towards less costly outcomes, the higher the uncertainty about the target location was. This interplay between estimation uncertainty and movement cost is consistent with a risk-sensitive decision criterion that takes model uncertainty into account.

## Introduction

Biological organisms have evolved to succeed in environments with considerable uncertainty [1]. One important way of dealing with uncertainty is to develop models of the environment and to form beliefs for prediction. Bayesian statistics provides a powerful and unifying framework to deal with uncertainty not only in the cognitive domain, but also in sensorimotor tasks [2]. Previous studies have shown that sensorimotor integration in uncertain environments is consistent with Bayesian integration by weighing prior expectations and sensory evidence according to their reliability [3]–[5]. In particular, it has been shown that the nervous system is able to extract the statistics of variable environments and to incorporate this information by modifying prior beliefs during the process of learning [6]. The same formalism can also be used to describe the weighing of information stemming from different sensory modalities with different reliability, for example, when integrating visual and haptic information. A number of previous studies have shown that such multi-modal integration in sensorimotor tasks is also in quantitative agreement with Bayesian statistics [7]–[9].

More generally, internal models are thought to play an important role during sensorimotor processing, for example, to predict sensory consequences of one's actions and to estimate the state of body parts from noisy sensory feedback [10]–[12]. For example, it has been shown that such estimation is consistent with Kalman filtering, a particular form of Bayesian updating, when subjects had to point to where they believed their hand was after making reaching movements in the dark [10]. As a generalization of this, Bayesian updating is also used as a module for estimation in optimal feedback control models [13]–[16] that have successfully explained a wide range of motor behaviors such as variability pattern [13], the response to of bimanual movements to perturbations [17], [18], adaptation to novel tasks [19]–[21] and complex object manipulation [22].

Bayes optimal decision-makers are, however, risk-neutral in the sense that they weigh all possibilities based on prior expectation and sensory evidence when they choose the action with highest expected value. In contrast, a risk-sensitive decision-maker also considers model uncertainty [23]. Intuitively, model uncertainty implies that the probabilistic Bayesian model is only trusted to some extent and that deviations from this model are possible towards worst case outcomes (risk-averse) or towards best case outcomes (risk-seeking)— especially if the predictions of the model are imprecise. This model uncertainty leads to an interesting interplay and biasing of estimation and control processes in risk-sensitive decision-makers [24]–[26]. Consider, for example, a goal keeper that tries to catch a ball flying towards the edge of the goal. Not only will he combine his prior beliefs about velocity, direction, etc. with his sensory evidence, but he will also consider the fact that there are quite different costs depending on which side of the goalpost the ball will most likely end up. In other real-life situations the implications of risk-sensitive estimation could even be more serious, for example when considering evidence for low-probability events like the possibility of a rare disease given some symptoms or the possibility of an aeroplane or a space rocket crashing given a malfunction signal from a noisy detector [27].

Recently, risk-sensitivity has been shown to be an important determinant of motor behavior [28]–[31]. The main finding of these studies was that subjects choose their motor commands not only to optimize the expectation value of some performance criterion, but that they are also sensitive to the variability of the achieved performance measure, which can lead to increased control gains [28], increased (or decreased) hitting velocities [30] and acceptance of decreased mean effort [31] in environments where performance is highly variable. However, there is an important aspect of risk-sensitivity that these previous studies have not considered: risk-sensitivity does not only affect the control process, but also the estimation process in uncertain environments with latent task variables that are not directly observable [24]. In uncertain environments with latent variables risk-sensitivity leads to effects of model uncertainty, whereby estimation can become biased by the costs that are involved in the control process and control can become biased by the uncertainty of the estimator [23]. Crucially, none of the previous studies on risk-sensitivity contained any latent variables. To investigate the effects of risk-sensitivity on the estimation process, we therefore designed a sensorimotor experiment that not only contained a latent variable that needed to be estimated, but we also introduced a cost that was associated with subjects' responses. This way we could test whether subjects would exhibit characteristic risk-sensitive biases.

## Results

**Figure 1:**
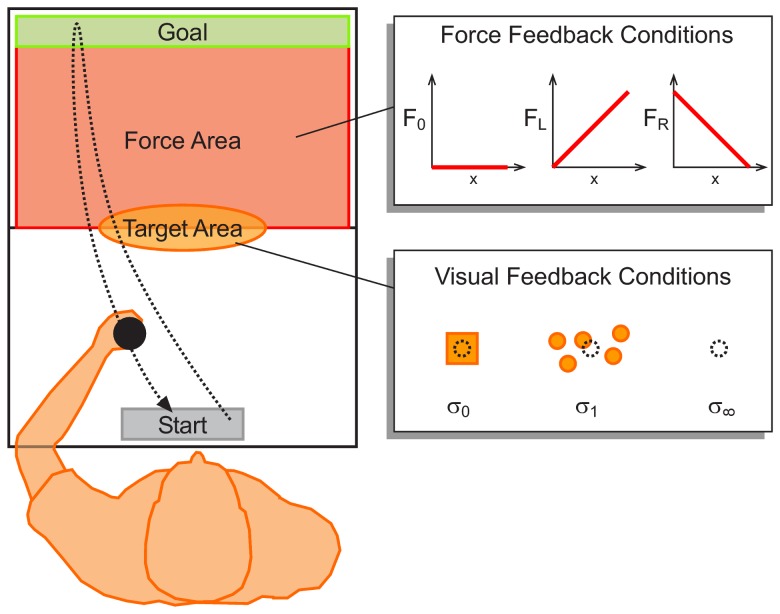
Experimental setup. Subjects move from a start bar to a goal bar and have to hit a target halfway in the reaching movement. In each trial the lateral position of the target was randomly drawn from a Gaussian distribution. The reliability of the visual feedback of the target position was manipulated, such that each trial belonged to one of three feedback conditions: 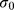, 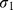 or 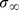. Furthermore, we imposed three different force functions (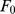, 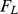 and 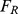) in the force area, where the force depended on the presumed target position as they indicated it by their forward movement. Screenshots of the actual display can be found in Text S1.

Subjects had to hit a target halfway in a reaching movement to a goal bar by controlling a cursor representing their hand position in a virtual reality set-up (Figure 1). In each trial the lateral position of the target was randomly drawn from a Gaussian distribution. However, the reliability of the visual feedback of the target position was manipulated, such that each trial belonged to one of three feedback conditions: 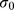, 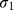 or 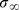. In the 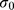-condition the target position was displayed clearly and throughout the trial, corresponding to full information and (practically) zero uncertainty. In the 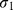-condition only blurry feedback was provided by displaying a short flash of a Gaussian cloud centered around the target. In the 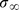-condition no feedback was provided. Naturally, the probability of hitting the target decreased with increasing feedback uncertainty— compare Figure S1 in Text S1. In this setup, the lateral target position constitutes a latent variable that needs to be estimated in every trial from noisy feedback. The aim is to study subjects' sensorimotor integration with respect to this latent variable and to study their susceptibility to risk-sensitive distortions.

**Figure 2:**
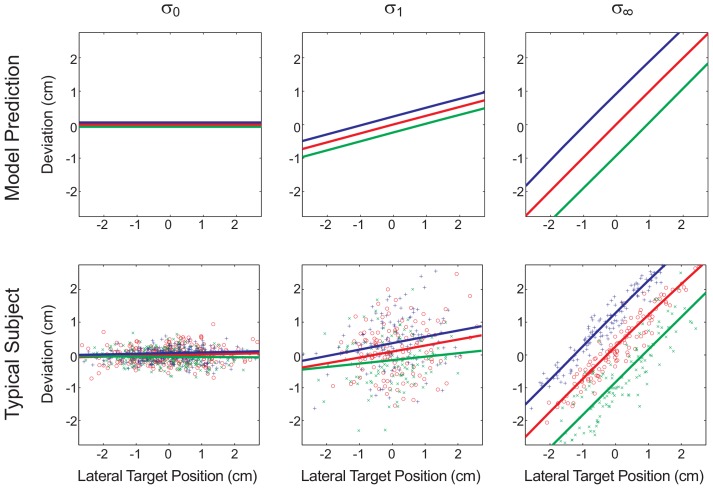
Lateral deviation from target as a function of target position in a risk-sensitive model (top row) and in a typical subject (bottom row). The three columns correspond to the three levels of uncertainty of the target feedback (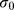, 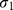 and 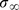). Each panel compares the three different force conditions 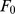 (red), 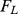 (green) and 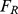 (blue). The model predicts that higher levels of uncertainty are associated with higher slopes and that higher forces are associated with shifts in the intercept that are proportional to the uncertainty.

Previous studies have shown that human sensorimotor integration of feedback information with varying degrees of reliability can be understood by Bayesian models [4]. In particular, it has been shown that subjects rely more on their prior information when the quality of their sensory feedback gets worse. This can be seen in Figure 2 which shows a typical subject's lateral deviation from the target as a function of the target position (red lines). In the full feedback condition (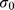) the lateral deviation was close to zero, as subjects could see the target clearly. In contrast, in the no-feedback condition (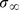) subjects had to rely on their prior about the target position and should ideally move through the point of maximum prior probability— which is zero in our case, such that the lateral deviation as a function of the target position is described by the identity line. The subject's behavior in the third panel of Figure 2 conforms to this prediction. Furthermore, the model predicts that in the 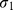-condition subjects should mix prior beliefs with sensory feedback, leading to an intermediate slope for the lateral deviation. We also found this effect in our subjects as displayed in the second panel of Figure 2. In summary, when comparing the red lines of the three panels of Figure 2, it can be seen that the slope of the lateral deviation increases with the uncertainty, which is exactly what previous studies have reported [4].

To investigate effects of risk-sensitivity we introduced a force landscape that assigned different costs to subjects' responses. The force landscape was given by a viscous force in the forward-backward direction during the second half of the movement between target and goal bar— this is indicated as the red force area in Figure 1. We imposed three different force functions (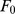, 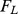 and 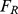) that were presented consecutively to subjects in three blocks of 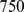 trials each. The 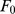-function was applied in the first block and corresponded to a zero force condition. The force 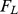 (“easy left”) was presented in the second block and corresponded to a linear function that increased from left to right. Therefore, pointing to a target position on the left required less effort than pointing to a target position on the right of the center of the target distribution. Finally, the force 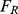 (“easy right”) was presented in the last block and corresponded to a linear function that decreased from left to right— see Methods for details.

Assigning cost to subjects' reponses predicts an interesting interaction between uncertainty and cost for a risk-sensitive decision-maker. In the absence of uncertainty (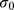-condition) there is no risk and a risk-sensitive decision-maker will produce the same behavior as a risk-neutral estimator that is independent of the imposed cost. However, in the presence of uncertainty, there is risk involved and a risk-sensitive decision-maker will bias its behavior based on cost. Having uncertainty about the target position implies that a risk-sensitive decision-maker has to consider a range of possible target positions and essentially “hopes” that the target is in one of the possible positions that requires less effort. In the case of linear force functions this “bias” translates into a parallel shift of the line that describes subjects' lateral deviation. The magnitude of the shift depends on the uncertainty of the target position, the cost of the presumed target position and subjects' risk-sensitivity. This prediction can be seen in Figure 2.

When reaching for the target, subjects had to combine prior information about the distribution of target positions, visual feedback and the cost of the pointing movement. We examined how they combined these three factors in the following way. For each force block (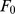, 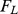 and 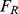) we conducted three linear regressions corresponding to the three feedback conditions (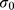, 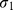 or 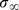). In each case we regressed the lateral deviation of subjects' pointing movement against the true target position and determined slope and intercept of this line. According to the model predictions in Figure 2, the slope should only depend on the uncertainty of the feedback independently of the force condition, whereas the intercept should depend on both the cost given by the force and the uncertainty given by the feedback condition.

**Figure 3:**
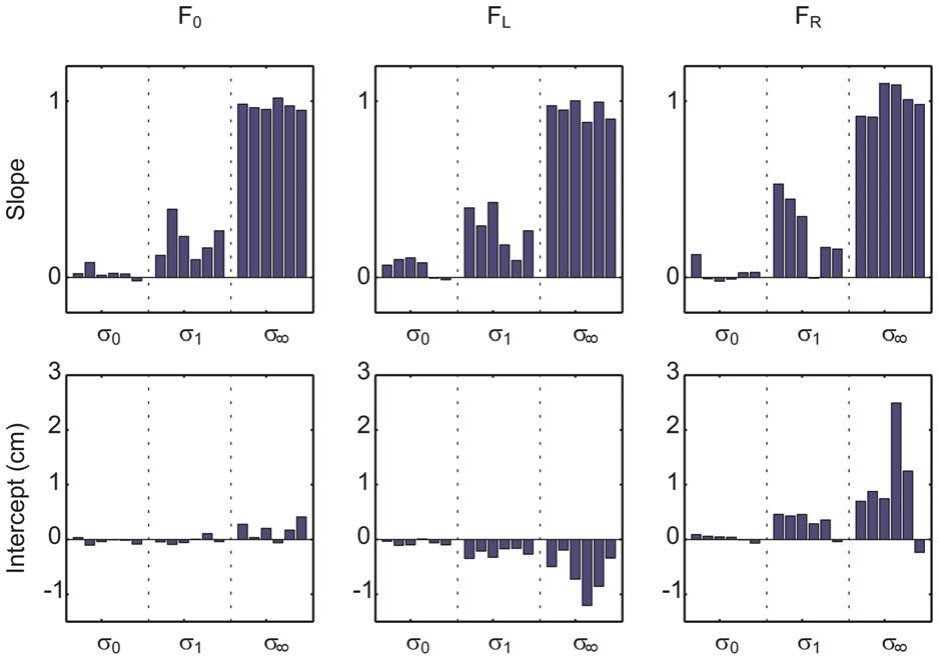
Slopes (top row) and intercepts (bottom row) of linear regression for all subjects. Linear regression was performed as in Figure 2. The three columns correspond to the three different force conditions 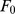, 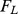 and 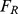. The three different feedback conditions 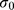, 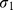 and 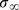 are displayed within each panel. It can be seen that the slope increases with increasing uncertainty. The intercepts are modulated by both uncertainty and force condition.

The slopes and intercepts fitted to every subject are shown in Figure 3. In the upper panels of Figure 3, one can see that the slopes describing subjects' lateral deviation increased with higher levels of uncertainty within each force block. This is in line with the prediction and reproduces previous findings. Moreover, in accordance with the prediction from Figure 2, this slope increase was not affected by the force condition. To assess the statistical significance of this result we conducted a repeated-measures two-way ANOVA with force and uncertainty as factors. We found that the uncertainty had a significant effect on the slope (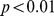), whereas the effect of force was not significant (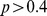).

In the lower panels of Figure 3, one can see subjects' intercepts that describe their mean lateral deviations from a reference target located in the center of the workspace (zero position). In accordance with the prediction from Figure 2, our ANOVA revealed that intercepts were affected by both uncertainty (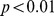) and force condition (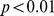). In the no-force condition the intercepts are close to zero for all uncertainty levels, as subjects have no incentive to deviate from an unbiased Bayesian estimate. In the force conditions 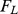, we found that the intercepts become increasingly negative with growing uncertainty. This means that subjects' behavior was biased towards the left, as target positions on this side were associated with lower costs. Compared to the no-force condition, subjects deviated on average 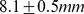 more to the left in the no-feedback condition and 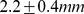 more to the left in the 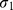-condition. Similarly, in the 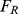 force condition, we found that intercepts increased with growing uncertainty reflecting a low-cost bias towards the right side of the workspace. Compared to the no-force condition, subjects on average deviated 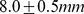 more to the right in the no-feedback condition and 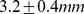 more to the right in the 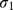-condition. All subjects but one exhibited this bias pattern— compare Figure 3.

Importantly, the model of risk-sensitive decision-making not only predicts a fixed bias— which is what a Bayes optimal decision-making model would predict— , but a modulation of bias and uncertainty, such that the bias increases with the amount of uncertainty and vanishes in the limit when uncertainty is absent. In accordance with this prediction, we found that the mean lateral deviations from the center of the target in the 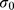-condition are negligible in all force conditions. The exact values of the mean lateral deviations were 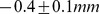 in the 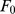-condition, 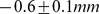 in the 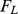-condition, and 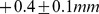 in the 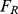-condition— all well within the target halfwidth of 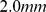. Similarly, the lateral deviations from the center of the starting position at the beginning of the trial was not significantly different between the groups (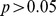, repeated measures one-way ANOVA). The exact values of the mean lateral deviations were 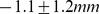 in the 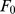-condition, 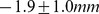 in the 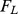-condition, and 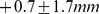 in the 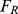-condition— all well within the target halfwidth of 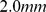. In summary, these results suggests that subjects did not simply avoid high costs, but that their behavior was determined by an interplay of uncertainty and cost as predicted by a risk-sensitive decision-making process.

## Discussion

In our study we examined the effects of risk-sensitivity on sensorimotor integration. In line with previous studies, we found that information integration was consistent with a Bayes optimal decision-maker as long as subjects' responses were cost-neutral [4]. However, once we introduced a cost that was associated with subjects' responses, subjects started to bias their behavior when faced with uncertain feedback. Importantly, subjects did not simply minimize their effort, but they modulated their behavior based on an interplay between cost and uncertainty. In particular, we found that the higher the uncertainty, the higher the bias. When sensory feedback was unambiguous— i.e. in the (near) absence of uncertainty— this bias vanished. This is in accordance with the predictions of a risk-sensitive decision-making process, but violates risk-neutral Bayes optimal integration.

Previous studies have found that risk-sensitivity is an important determinant of motor behavior [29]. The main finding of these studies was that subjects not only optimize their expectation of success, but also take the performance variability into account. For example, a basket ball player choosing between throwing a three with a 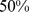 success rate and throwing a two with a 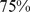 success rate would prefer the first option if risk-seeking, the second option if risk-averse, and he would be indifferent if risk-neutral. These previous studies have found that risk-sensitive motor behavior can be accounted for by a mean-variance trade-off [31] that affects control gains and the speed-accuracy trade-off when performance success becomes more variable [28], [30]. Importantly, the effects of risk-sensitivity on the estimation process could not be investigated in these previous studies, because they did not contain any latent variables that would have required estimation.

The differential effects of risk-sensitivity on control and estimation can be readily inspected in the case of risk-sensitive control of linear systems with quadratic costs and Gaussian noise— sometimes abbreviated to risk-sensitive LQG control [24]. The standard LQG control that has often been used in optimal feedback control models of motor behavior [13] can be derived as a special case of the risk-sensitive LQG control in the limit of vanishing risk-sensitivity. Importantly, in risk-neutral LQG controllers the estimation and control processes can be separated such that the solution to the estimation problem is given by the Kalman filter and the solution to the LQ control is given by the solution of the Riccati equation. The overall solution to the LQG system is then simply given by the LQ optimal controller where all directly observed variables are replaced by their estimates from the Kalman filter. In summary, in the risk-neutral case the estimates are obtained independent of the controls, and the control law is obtained independently of the estimation process.

Effects of risk-sensitivity in optimal feedback control have been previously studied in [28], however in the absence of observation noise— i.e. in the absence of a latent variable. In this case the solution to the risk-sensitive LQG control problem is given by the solution of a modified Riccati equation. Nagengast et al. [28] studied effects of this modification of the control process, for example, the change in control gain in response to increased process noise that determined the Brownian motion of a virtual ball. Crucially, however, the observation noise was entirely negligible compared to the process noise in this task, so effects of risk-sensitive estimation did not play any role in this experiment.

In the presence of observation noise, i.e. in the presence of a latent variable, estimation and control processes are no longer independent, but they have interesting interconnections between them that are absent in risk-neutral systems [24]. There is a modified risk-sensitive Kalman filter that depends on control costs, a distortion of the Riccati equation depending on the uncertainty, and a distorted certainty-equivalent that is the value that is reported from the modified Kalman filter to the controller. In our experiment we introduced a force as a cost to subjects' responses when they report the latent variable, that is the presumed target position. We can model this process in terms of risk-sensitive LQG control as follows— compare Text S1. The Kalman filter estimate of the target position is unbiased, yielding a Bayesian estimate 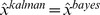. However, the certainty-equivalent value 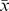 that is conveyed to the controller is a distortion of the Kalman filter estimate, that is 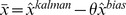. The control is given by 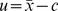, where 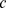 is a constant that trades off the importance of reaching the target against the strength of the field. If the field becomes excessively strong, at some point the optimal controller would simply ignore the target and point to the position with lowest cost. As we did not see a significant constant shift across the three uncertainty conditions in our experiment, this constant— which is a free parameter— was very small— which means that subjects cared much more about hitting the target than the force.

Most importantly, we can rule out a risk-neutral account of our experiment, since the observed bias term depends on the risk-sensitivity. In particular, we can rule out that subjects simply trade off the expected loss of missing the target against the cost of the force. Ultimately, it is the risk-sensitive cost function that considers higher order moments of the expected costs that leads to a coupling between estimation and control in risk-sensitive decision-makers that do inference over latent variables. This coupling predicts exactly the interplay between uncertainty and force that we observed in our experiment, namely that subjects apparently cared less and less about hitting the target when the prediction about the target location was imprecise. A risk-sensitive subject therefore allows for deviations as if following the maxim “If I'm not going to get the target anyway— because the uncertainty is high— , why not miss it in the less costly fashion”.

Another possible explanation could be that subjects care less about hitting the target, not because of the uncertainty of where it is, but because the hitting probabilities are low. This would predict that if subjects attempted to hit smaller targets that have lower hitting probabilities, but no associated uncertainty with respect to location, they should exhibit the same kind of bias. This is however unlikely to be the case, as subjects would explicitly have to violate the task description and move away from a target that they can clearly see, just because it is small.

Another related question is also whether biases occurred not due to uncertainty about the target position, but due to imprecision about performance success in no-feedback trials. Like previous studies on Bayesian integration [4] we assumed that the statistics applicable to no-feedback trials are learned in trials that have full feedback. For example, Kording and Wolpert [4] did not show any terminal feedback after completing no-feedback trials, but terminal feedback was only shown in full feedback trials, so as to probe the inference process without giving subjects the possibility of learning a mapping in the no-feedback trials. In our study we additionally introduced binary auditory feedback after each trial to indicate whether the target was hit or not. This auditory feedback was also provided after no-feedback trials to give subjects an idea about their success rate and to indicate that there really is a target even though it cannot be seen, but without giving them the explicit possibility of learning a mapping. However, we cannot exclude the possibility that revealing the true target position with respect to the subjects' response *after* the trial could have reduced the observed bias. On the one hand, revealing the true target position in these trials would not provide any new statistics about the target location since these were the same in all trial types. On the other hand, highlighting subjects' “misjudgements” under the supervision of an eager experimenter might well lead to a reduction in bias. However, this might also be regarded as introducing an extra cost. Therefore, the imprecision of performance feedback might influence subjects' responses, but this is not necessarily in disarray with the predictions of a risk-sensitive decision-making process.

In the future it could also be interesting to study risk-sensitive models in the context of “wishful thinking” when people overestimate their own abilities [32]–[36]. What makes risk-sensitivity especially interesting in the context of Bayesian inference is that it has also been related to model uncertainty [23]. Model uncertainty allows a decision-maker who has a probabilistic model of the environment to deviate from this model if he trusts this model only to a limited extent. In particular, an infinitely pessimistic decision-maker would disregard the probabilistic model entirely and only focus on worst-case outcomes. Since all models are typically prone to error at some precision, taking into account model uncertainty is a crucial aspect of estimation and control.

## Methods

### Ethics Statement

All experimental procedures were approved by the ethics committee of the medical faculty at the university of Tübingen.

### Subjects

Two female and four male subjects from the Tübingen University student population participated in this experiment after giving informed consent. Participants were paid the local standard rate of 8 Euros per hour for their participation.

### Materials

The experiment was conducted using a vBOT robotic manipulandum [37]. Participants controlled the vBOT handle in the horizontal plane. Movement position and velocity were recorded at a rate of 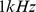. A planar virtual reality projection system was used to overlay images into the plane of movement of the vBOT handle.

### Experimental Procedure

Subjects performed reaching movements from a start bar (gray rectangle, width 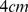, height 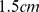) to a goal bar (green rectangle, width 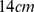, height 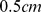) 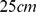 away by moving a cursor (red circle, 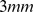 radius) representing their hand position— compare Figure 1. The hand position was represented veridically at all times. Subjects could start anywhere from within the start bar and they were told to hit a yellow target that would appear midway during the forward movement to the green bar. When placing the cursor on the start bar, participants heard a beep that informed them to move. At the same time the target appeared midway at a distance of 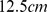 from the start bar with a lateral displacement drawn from a Gaussian distribution with zero mean and standard deviation 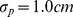. Movements had to be completed within 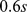.

In each trial the target position was displayed under one out of three possible feedback conditions (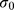, 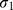, 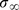) selected randomly with relative frequencies of (2,1,1) respectively. In the 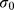-condition, the target was displayed during the whole trial as a small rectangle of 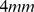 width. The displayed height of the target was 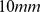, but only relevant for visualization purposes without consequence for the hitting probability. In the 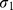-condition, five small circles (radius 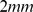) were drawn each trial from a two-dimensional Gaussian distribution (mean 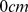, standard deviation 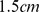) and shown for 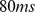 at the beginning of the trial. No feedback was provided in the 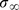-condition. In all three conditions subjects had to make a choice in the lateral position 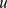 when they were halfway in the movement (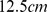 from the start bar) in order to indicate their belief about the target position. Halfway into the movement they also received auditory feedback, which was a high frequency beep if they hit the target or a low frequency beep if they failed to do so. Another beep of the same frequency informed them when they reached the goal bar.

Between the target and the goal bar subjects entered a “force zone” in which they experienced a viscous force 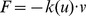 that made movements more strenuous. The viscous force was applied in the forward-backward direction and was proportional to the forward or backward velocity 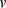. The force was also applied in the force zone while subjects returned to the start position to initiate the next trial. The strength 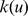 of the force depended only on subjects' movement position 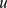 halfway into the movement (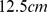 from the start bar). To allow for a smooth transition from the no-force zone to the force zone the viscous force was ramped up linearly over the first quarter of the force zone and similarly ramped down during the backward movement. There were three force conditions: 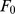, 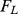 and 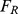. In the 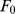 condition there was no force, that is 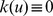. In the 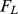 condition the strength 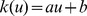 was a linear function with 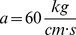 and 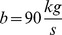, such that it increased linearly from left 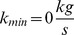 to right 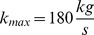 over a 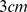 range centered around the mean of the target distribution. In the 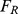 condition the slope was simply inverted to obtain a linear function with 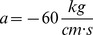 and 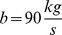 that increased linearly from right 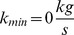 to left 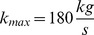 over the same 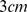 range.

The experiment consisted of 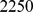 trials in total and was subdivided in three blocks of 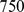 trials each corresponding to the three force conditions 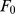, 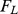 and 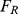. In every block of 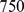 trials only the last 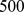 were used for analysis, as movement variability in 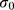-trials had then stabilized— compare Figure S2 in Text S1.

### Risk-neutral Decision-maker

Each trial a target with lateral position 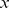 is drawn from a Gaussian distribution with mean zero and standard deviation 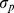. Subjects receive noisy sensory feedback about the target position given by the observation 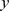. We model this noisy feedback by another Gaussian distribution with mean 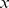 and standard deviation given by 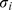 where 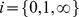. Subjects report their estimate of the presumed target position by a controlling the lateral response 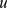. In our experiments subjects' response incurred a cost of the form 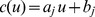 with 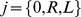. The cost 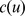 models the experimental viscosity function 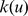 described in the Experimental Procedures. The parameters 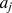 and 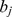 depend on the force condition, where 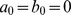 in the 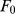-condition and 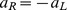 and 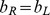 in the other force conditions. The risk-neutral Bayes optimal decision-maker that trades off a quadratic cost for the target hit and the linear response cost is then given by
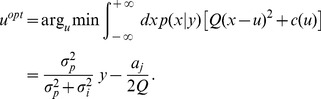
Importantly, the bias 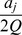 does not depend on the uncertainty level and simply formalizes a trade-off between the importance of reaching the target Q and the strength of the force 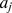. Since we did not observe a constant bias in the 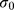-condition in our experiment, it is 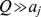, that means we can safely neglect this term.

### Risk-sensitive Decision-maker

A risk-sensitive decision-maker with risk-sensitivity parameter 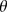 optimizes the following risk-sensitive stress function [24], [25]

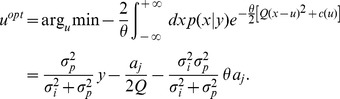
(1) Again the second term is constant and can be neglected, as 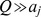 in our experiment. The important part is the third term that incorporates an interaction between cost 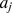 and the uncertainty given by 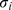 and 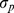. This predicts increasing biases for increasing uncertainty. In the limit 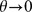 the risk-sensitive decision-maker becomes the risk-neutral decision-maker.

## Supporting Information

Text S1 Supplementary material including the supplementary Figures S1 and S2.
(PDF)
