# Supplementary material for: Risk-Sensitivity in Bayesian Sensorimotor Integration
Source: PLoS Comput Biol. 2012 Sep 27;8(9):e1002698. doi: 10.1371/journal.pcbi.1002698 (PMC3459842; doi:10.1371/journal.pcbi.1002698)
Supplement: Text S1 — Supplementary material including the supplementary Figures S1 and S2. (PDF) [file pcbi.1002698.s001.pdf]

Supplementary Information: Risk-sensitivity in Bayesian sensorimotor integration

Jordi Grau-Moya, Pedro A. Ortega, Daniel A. Braun

Risk-sensitive Linear Quadratic Gaussian Control

In order to fit with the formalism proposed in [1], we can translate our experiment into a 3-step system with the following scalar variables

$$\begin{aligned} x_1 &= x_0 + \epsilon \\ y_1 &= x_0 + \eta_\infty \\ c_1 &= 0 \\ \\ x_2 &= x_1 \\ y_2 &= x_1 + \eta \\ c_2 &= ku_2 \\ \\ x_3 &= x_2 - u_2 \\ y_3 &= x_2 + \eta_\infty \\ c_3 &= x_3 Q x_3. \end{aligned}$$

The integral cost is given by $J = \sum_{t=1}^{3} c_t = x_3 Q x_3 + k u_2$, where the first term enforces that the difference between the control signal and the target position is minimized, and the second term is a linear cost that models the force cost that we imposed in our experiment. The risk-sensitive *stress* function $\gamma(\theta)$ that is to be minimized is given by

$$\gamma(\theta) = -\frac{2}{\theta} \log \mathbb{E}\left[e^{-\frac{\theta}{2}J}\right].$$

The system evolves as follows:

- In the first time step, the target position x_1 is drawn from a Gaussian distribution with mean x_0 and variance σ_n^2, that is $\epsilon \sim \mathcal{N}(0, \sigma_n^2)$. The mean is assumed to be known precisely, that is $\hat{x}_0 = x_0$ with variance $V_0 = 0$. No observation is made, or formally $\eta_\infty \sim \mathcal{N}(0, \infty)$. No control is applied, that is $u_0 = 0$.
- In the second time step, a noisy observation y_2 of the target position x_1 is made. The observation noise is additive and drawn from a Gaussian distribution with $\eta \sim \mathcal{N}(0, \sigma_m^2)$. The target position does not change during the observation, that is $x_2 = x_1$. No control is applied, that is $u_1 = 0$.
- In the third time step, a control command u_2 can be applied to minimize the quadratic cost $(x_2 - u_2)Q(x_2 - u_2)$, which implies that the control should match the target position. No further observations are made.

Minimizing the stress function $\gamma(\theta)$ can be achieved by computing the *past stress* $P_t(x_t)$ for estimation and the *future stress* $F(x_t)$ for control. Whittle [1] derived the following recursions for past and future stress:

$$\begin{aligned} P_{t+1}(x_{t+1}) &= \text{ext}_{x+t}\left[P_t(x_t) + c_t + \frac{1}{\theta}(n_t + m_t)\right] \\ F_t(x_t) &= \min_{u_t} \text{ext}_{x_{t+1}}\left[c_t + \frac{1}{\theta} n_t + F_{t+1}(x_{t+1})\right], \end{aligned}$$

where "ext" indicates min or max depending on the sign of θ and the shorthands n_t and m_t are given by

$$
\begin{aligned}
n_t &= (x_{t+1} - x_t - u_t)^2 \, \sigma_n^{-2} \\
m_t &= (y_{t+1} - x_t)^2 \, \sigma_m^{-2}.
\end{aligned}
$$

Whittle [1] could show that the optimal control u_t^{opt} that minimizes $\gamma(\theta)$ can be computed by finding the u_t^* that minimizes the future stress $F_t(x_t)$ and finding the certainty-equivalent $\bar{x}_t$ that extremizes the combined stress $P_t(x_t) + F_t(x_t)$, such that $u_t^{\text{opt}} = u_t^*(\bar{x}_t, t)$. This establishes a risk-sensitive version of certainty-equivalence, where minimizing the past stress leads to a risk-sensitive version of the Kalman filter with recursively updated estimates $\hat{x}_t$ (mean) and V_t (variance) representing a Gaussian belief. For our system equations this results in the following:

Initialization:

$$
\begin{aligned}
P(x_0) &= 0 \\
\hat{x}_0 &= x_0 \\
V_0 &= 0
\end{aligned}
$$

First time step:

$$
\begin{aligned}
P(x_1) &= \frac{1}{\theta}(x_1 - \hat{x}_0)^2 \sigma_n^{-2} \\
\hat{x}_1 &= \hat{x}_0 \\
V_1 &= \sigma_n^2
\end{aligned}
$$

Second time step:

$$
\begin{aligned}
P(x_2) &= \frac{1}{\theta}(x_2 - \hat{x}_0)^2 \sigma_n^{-2} + \frac{1}{\theta}(y_2 - x_2)^2 \sigma_m^{-2} \\
\hat{x}_2 &= \frac{\sigma_n^{-2}\hat{x}_0 + \sigma_m^{-2} y_2}{\sigma_n^{-2} + \sigma_m^{-2}} \\
V_2 &= \left(\sigma_n^{-2} + \sigma_n^{-2}\right)^{-1}
\end{aligned}
$$

$$
\begin{aligned}
F(x_2) &= \min_{u_2} \left\{ Q(x_2 - u_2)^2 + k u_2 \right\} = 0 \\
u_2^* &= x_2 - \frac{k}{2Q} \\
\bar{x}_2 &= \frac{\sigma_n^{-2}\hat{x}_0 + \sigma_m^{-2} y_2 - \frac{1}{2}\theta k}{\sigma_m^{-2} + \sigma_n^{-2}} \\
u_2^{\text{opt}} &= \bar{x}_2 - \frac{k}{2Q} = \hat{x}_2 - \frac{k\theta}{2\sigma_n^{-2}\sigma_m^{-2}} - \frac{k}{2Q}
\end{aligned}
$$

The controller u_2^{opt} is identical to the controller derived in the main manuscript. By translating it into the format in [1], the individual terms can be matched to the Kalman filter, the certainty-equivalent estimate and the certainty-equivalent control command.

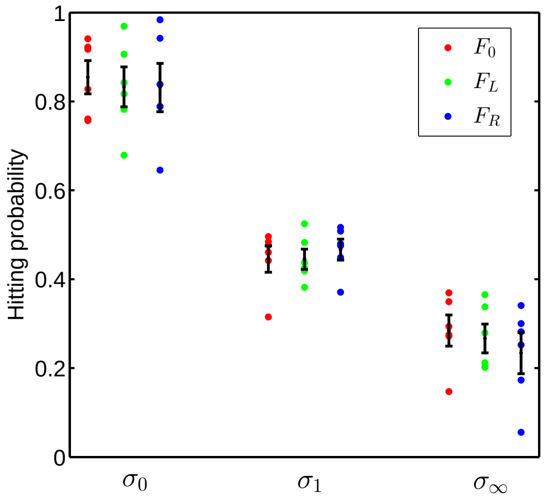

Figure S 1. Hitting probabilities. Success probability of hitting the target for the three different feedback conditions (σ_0, σ_1 and σ_∞) and the three different force conditions F_0 (red), F_L (green) and F_R (blue). The hitting probability decreases with increasing feedback uncertainty.

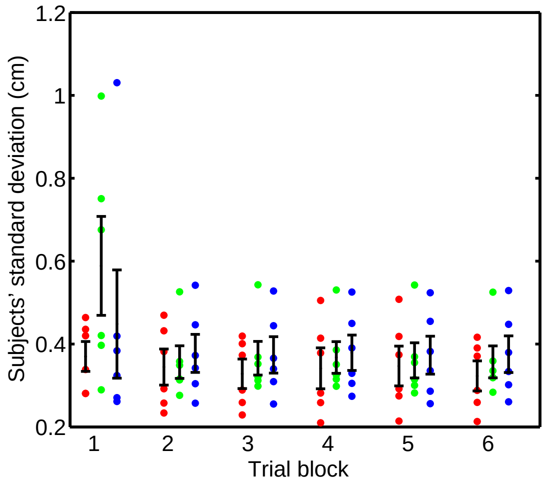

Figure S 2. Movement variability. Standard deviation of hitting movements in trials of the σ_0-condition over blocks of 125 trials. Since variability was increased for some subjects in the first block, we only analyzed the last 500 trials of each force condition in the experiment.

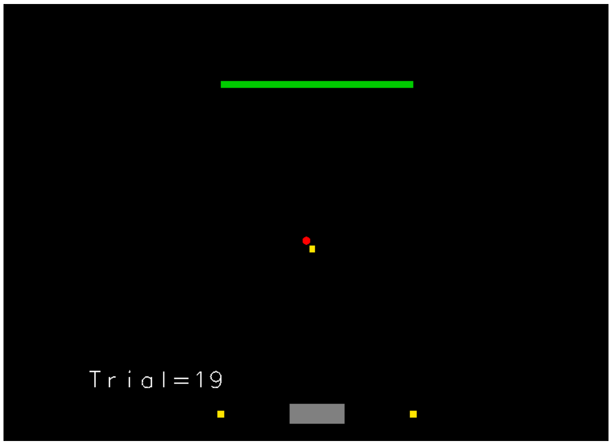

Figure S 3. Screenshot of the display for the σ_0-condition.

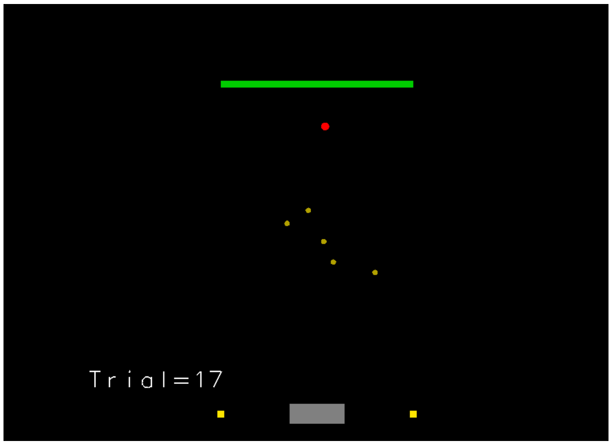

Figure S 4. Screenshot of the display for the σ_1-condition.

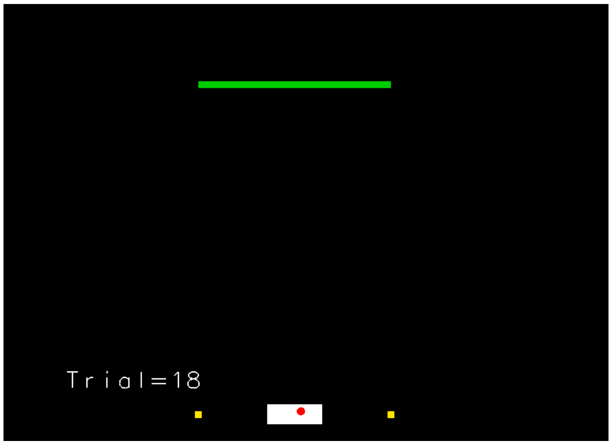

Figure S 5. Screenshot of the display for the σ_∞-condition.

References

1. Whittle P (1981) Risk-sensitive linear/quadratic/Gaussian control. Advances in Applied Probability 13: 764–777.
